# Treatment of supraglottic squamous cell carcinoma with advanced technologies: observational prospective evaluation of oncological outcomes, functional outcomes, quality of life and cost-effectiveness (SUPRA-QoL)

**DOI:** 10.1186/s12885-023-10953-9

**Published:** 2023-06-01

**Authors:** S. Hassid, B. Krug, S. Deheneffe, J-F. Daisne, G. Delahaut, G. Lawson, R. Crott, S. Van der Vorst

**Affiliations:** 1 Department of Otorhinolaryngology, UCLouvain, CHU UCL Namur (Site Godinne), Head & Neck Surgery, Yvoir, Belgium; 2 Department of Nuclear Medicine, UCLouvain, CHU UCL Namur (Site Godinne), Yvoir, Belgium; 3 Department of Radiotherapy, UCLouvain, CHU UCL Namur (Site St-Elisabeth), Namur, Belgium; 4 Department of Radiation-Oncology, Catholic University of Leuven (KU Leuven), University Hospital UZ Leuven, Louvain, Belgium; 5 Leuvens Kankerinstituut, Louvain, Belgium; 6 Health Economics Consultant, Formerly at Institute de Recherche Santé Et Société (IRSS)UCLouvain, Louvain, Belgium

**Keywords:** Head and neck cancer, Supraglottic, Squamous cell carcinoma, Radiotherapy, Transoral robotic surgery, Transoral Laser Microsurgery and Quality of life

## Abstract

**Background:**

Over the past decade, therapeutic options in head and neck supraglottic squamous cell carcinoma have constantly evolved. The classical total laryngectomy has been partially replaced by alternative organ- and function-sparing techniques with the same prognosis but less morbidity, such as Radiotherapy, Transoral Laser Microsurgery (TLM) and Trans-Oral Robotic Surgery (TORS). Up to now, a prospective comparison of these innovant techniques has not been conducted.

**Methods/design:**

We will conduct an original international multicentric prospective nonrandomized clinical trial to compare the efficacy between these treatments (Arm 1: Radiotherapy ± chemotherapy; Arm 2: TLM and Arm 3: TORS) with 4 classes of outcomes: quality of life (QoL), oncological outcomes, functional outcomes and economic resources. The population will include cT1-T2 /cN0-N1/M0 supraglottic squamous cell carcinoma. The primary outcome is a Clinical Dysphagia QoL evaluation assessed by the MD Anderson Dysphagia questionnaire. Secondary outcomes include others QoL evaluation, oncological and functional measures and cost parameters. The sample size needs to reach 36 patients per arm (total 108).

**Discussion:**

In the current literature, no prospective head-to-head trials are available to compare objectively these different treatments. With the increase of highly efficient treatments and the increase of oncological survival, it is imperative also to develop management strategies that optimize QoL and functional results. We will conduct this innovate prospective trial in order to obtain objective data in these two main issues.

**Trial registration:**

NCT05611515 posted on 10/11/2022 (clinicaltrial.fgov).

## Background

Head and Neck Squamous Cell Carcinoma (HNSCC) are the seventh most common cancer worldwide, with approximately 600.000 new cases diagnosed each year [1]. In Supraglottic Squamous Cell Carcinoma (SSCC), total laryngectomy has been the mainstay treatment until the late 80 s’. However, laryngectomies are associated with an important loss of key function such as phonation and swallowing. More recently new organ preservation techniques have emerged, allowing better functional outcomes for patients.

First, partial open laryngectomy is associated to high stable local control rate (around 77%) [2–4] and offers the advantage of a functional larynx preservation rate in 80% of the patients [5, 6]. Due to the cervical access to the tumor, the recovery to safe swallowing takes time with the need of a transitory tracheostomy and a prolonged hospitalization time with parenteral intakes [7]. Aspiration pneumonia rate is around 6% [6].

Secondarily, the development of new radiation techniques (Intensity Modulated Radiotherapy, IMRT) and the concurrent administration of chemotherapy drugs allow better organ preservation and reduce tracheostomy rate [8–10]. The oncological outcomes remain stable, with same regional control of 70% at 5 years [11–13]. Aspiration rate (a dysphagia marker) remains in 14% of reported cases and edema with respiratory problems can occur [14]. In 16.8% of the cases a functional laryngectomy or permanent tracheostomy is required [14]. This can be explained by pharyngeal mucosa and constrictor muscles injury, as well as fibrosis and decreased pharyngeal peristalsis induced by radiation [15–17].

Thirdly, the development of new surgical instruments enables a Trans-oral approach with Laser Microsurgery (TLM). TLM gives a 2-year Overall Survival (OS) of 83% for pT1-T2 tumors, with a larynx preservation in 95% [18, 19]. However in that study, 13% of the patients needed a temporary tracheostomy and 3% kept a gastrostomy at 1 year [18]. The main limitation of TLM is the linear view of the microscope, as well as the laser beams, which requires a piecemeal resection of the tumor in difficult cases [20].

Subsequently, Trans-oral Robotic Surgery (TORS) emerged as an evolution of the TLM [21–24]. The main advantages are the 3D-high-quality camera and the dexterity improvement with 7 degrees of freedom articulated arms [25–27]. Some studies concerning supraglottic location reported a 2-year OS of 89% without the need of tracheostomy or gastrostomy [28, 29]. Patients started oral feeds at an average of 2 weeks [30].

Actually, the current standards of treatment are based on either surgery or radiotherapy. The treatment choice is center-, tumor- and patient-dependent with different side effect profiles and technical constraints [31, 32]. To the best of our knowledge, there is no head-to-head prospective comparison between TLM, TORS and radiotherapy. Moreover, in the available retrospective studies, functional data are often not comparable. In two meta-analyses, the oncological outcomes (OS and Disease-Specific Survival (DSS)) seem to be more favorable for TORS and TLM group (Odd Ratio (OR) 43% and 40%) compared to the radiation group, which is still the mainstay of treatment [14, 33]. Probably, the transoral approaches alone will confirm or even improve functional benefit. The authors concluded that prospective functional studies with objective measures are needed [14, 33].

Concerning the QoL of the patients treated for HNSCC, one prospective study has been published (“ORATOR study” [34]) while another is still ongoing (“Best-of European study”[35]). In the ORATOR study, patients treated with radiotherapy showed superior swallowing-related QOL scores at 1 year after treatment, although the difference did not represent a clinically meaningful change. Moreover, in the TORS subgroup, 71% patients were treated with adjuvant radio(chemo)therapy which makes the results difficult to interpret. However, these two studies investigate exclusively the outcomes of patients with oropharynx squamous cell carcinoma. There is currently no prospective study about the QoL of patients treated for a supraglottic cancer.

The main objective of this trial is to assess and compare objectively and prospectively the efficacy of different therapeutic approaches (radiotherapy ± chemotherapy, TLM and TORS) in SSCC patients according to 4 classes of outcomes; quality of life, oncological, functional and economic resources.

## Methods – design

We propose to carry out an observational non-randomized prospective clinical trial on patients diagnosed with an early SSCC where 3 subgroups are studied in parallel:

- Arm 1: Radiotherapy ± chemotherapy (RCT)
- Arm 2: Trans-oral Laser Microsurgery (TLM)
- Arm 3: Trans-Oral Robotic Surgery (TORS)

**Fig. 1:**
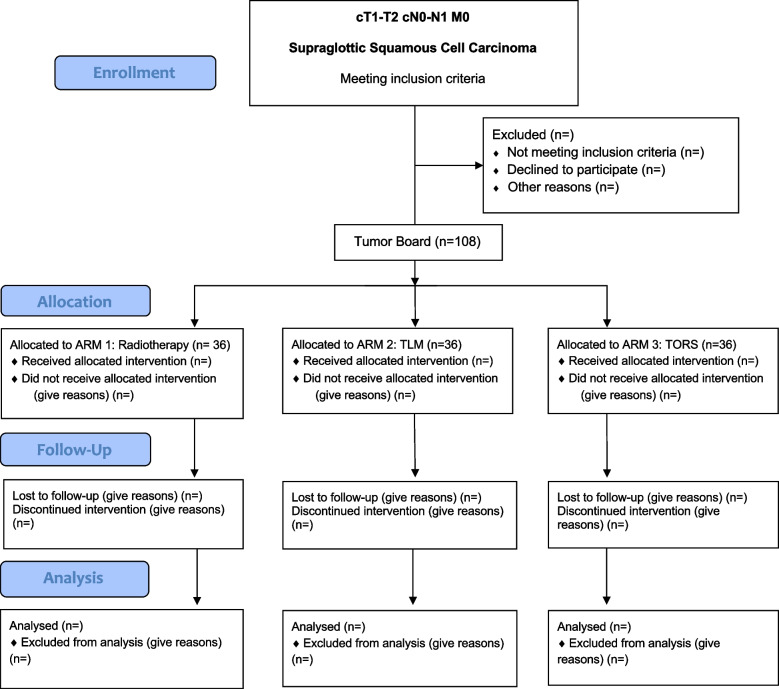
SPIRIT Flow Diagram

**Fig. 2:**
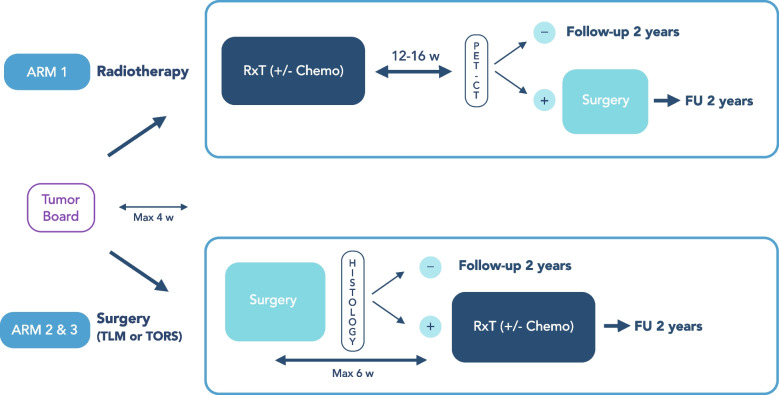
Trial Design

The inclusion will be multicentric and international. The treatment will be chosen by the local multidisciplinary tumor board (MTB) according to the primary cTNM-stage, the patient preferences and the local standard of care based on peer-reviewed international guidelines (Fig. 1, SPIRIT Flow Chart and Fig. 2, Trial Design). The guidelines to be followed are the National Comprehensive Cancer Network (NCCN) [31] or the European guidelines including the European Head and Neck Society (EHNS), the European Society of Medical Oncology (ESMO) and the European Society for RadioTherapy and Oncology (ESTRO) [32].

### Inclusion criteria

- Diagnosis of SSCC (with histological confirmation)
  - cT1-T2 / cN0-N1/ M0 according to 8.^th^ TNM classification (UICC/AJCC) [36]
  - WITH a MTB decision according to the NCCN or European guidelines [31, 32]
- Diagnostic imaging (Head and neck and pulmonary CT or PET/CT, ± IRM if needed) realized within 1 month before the study inclusion
-  ≥ 18 years old and able to provide an informed consent
- ECOG/WHO performance status ≤ 2 [37]

All participants must have a writing informed consent before their involvement.

### Exclusion criteria

- Previous radiotherapy ± chemotherapy treatment of the head and neck region
- Previous history of head and neck cancer within 5 years
- Prior invasive malignant disease unless disease-free for at least 5 years or more, with exception of non-melanoma skin cancer
- Non-supraglottic or unknown primary site
- Clinical and radiological signs of nodal extracapsular extension
- Significant trismus (maximum inter-incisal opening ≤ 35 mm)
- Pre-existing dysphagia not related to the cancer or the biopsy (from neurological disorders for example)
- Unable or unwilling to complete Quality of Life questionnaires
- Serious medical comorbidities or contraindication for surgery and/or radiation
- Pregnancy and lactation

### Primary outcome measure

Clinical Dysphagia QoL evaluation after treatment will be assessed using MD Anderson Dysphagia Index (MDADI) at 1 year [38, 39]. It contains 20 items; ranged in score from 0 to 100 (a high scale score represents a higher response level). Time Frame: baseline (before treatment), 3 – 6 – 9 – 12 – 18 – 24 months after treatment.

### Secondary outcome measures (Fig. 3)

**Fig. 3:**
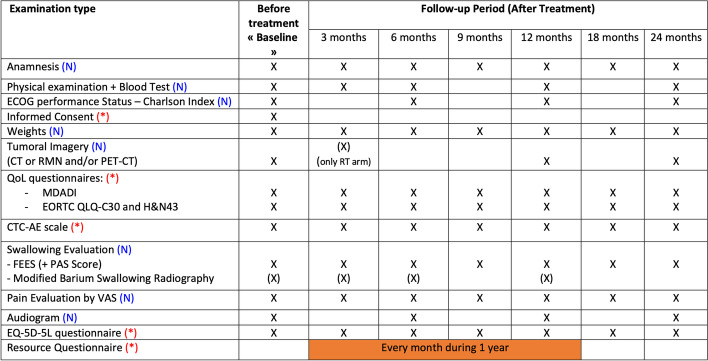
Examination planning

#### QoL measures

QoL will be evaluated with the validated European Organization for Research and Treatment of Cancer (EORTC) QLQ-C30 and H&N43 questionnaires ([40–42]. Time Frame: baseline (before treatment), 3 – 6 – 9 – 12 – 18 – 24 months after end of treatment (EOT).

#### Oncological measures

Overall survival, Disease specific survival, Disease-free survival, loco-regional and distant cumulative recurrence rate (%). Time Frame: 24 months from the start of treatment.

Histopathological finding for surgical arms: section margins status (R0/R1 and distance in mm) and P16 or HPV tumor status.

#### Functional measures

- Weight *(Kg).* Time Frame: 6 months before the diagnosis, baseline (before treatment), 1 – 3 – 6 – 9 – 12 – 18 – 24 months after treatment.
- CTC-AE score for early and late complications. Time Frame: baseline (before treatment), 1 – 3 – 6 – 9 – 12 – 18 – 24 months after treatment [43, 44].
- Naso-gastric Feeding tube and/or gastrostomy use *(duration in days).*
- Tracheostomy use (duration in days).
*-* Swallowing function by FEES evaluation (Fiberoptic Endoscopic Evaluation of Swallowing) objectively evaluated by the Penetration-aspiration Scale (PAS [45]). Time Frame: baseline (before treatment), 1 – 3 – 6 – 9 – 12 – 18 – 24 months after treatment.
- Treatment-related pain with Self-reported Visual Analogue Scale (VAS) and need for pain medication.
- Need of hospitalization (if yes, *duration in days*).
- Blood Test. Timeframe: Baseline (before treatment) – 3 – 6 – 12 – 24 months after treatments.
- ECOG performance status and Charlson Comorbidity Index [37, 46]. Timeframe: baseline (before treatment) – 6 – 12 – 24 months after treatment.

#### Cost measures

To set-up an economic analysis, we will collect resource data along this trial for the different treatment strategies and their complications through database analyses, chart review, patient diaries and EQ-5D-5L questionnaires [47, 48]. The follow-up starts just after the therapy (surgeries or radiation) and ends at 1-year post-intervention. The health care resource use items include acute and post-acute care, length of stay, medication use, outpatient and inpatient hospital visits, laboratory tests, radiology and medical professional visits. Resources used to treat complications will be recorded separately.

### Interventions

#### Arm 1: Radiotherapy ± chemotherapy (RCT)

Volumetric Modulated Arc Therapy (VMAT) with simultaneous Integrated Boost (SIB) will be used for all patients in this study.

For all cT1-T2N0 tumors, patients will be treated with moderately accelerated radiotherapy alone. Primary Gross Tumor Volume (GTVp) will be delineated according to the clinical examination and the available imaging. A 10 and a 5 mm margins (adapted to anatomical barriers) will be added to generate the Primary Clinical Target Volumes (CTVp) treated at prophylactic and radical (or therapeutic) doses, respectively (according to the current guidelines [49–51]):

- The therapeutic CTVp includes the tumor area and the high-risk location. It will be irradiated up to a total dose of 69 Gy in 30 fractions (2,3 Gy/fraction, 5 fractions a week during 6 weeks) or 70 Gy in 35 fractions (2,0 Gy/fraction, 6 fractions a week during 6 weeks).
- The prophylactic CTVp includes the elective mucosa. It will be irradiated up to a total dose of 54 Gy in 30 fractions (1,8 Gy/fraction, 5 fractions a week during 6 weeks) or 54,25 Gy in 35 fractions (1,55 Gy/fraction, 6 fractions a week during 6 weeks).

Elective CTVn must be selected and delineated according to the international recommendations and treated at prophylactic dose [50, 52].

All CTVs will be expanded by an isotropic 3–5 mm margin to generate the related Planning Target Volume (PTV). Margin may be cropped at level of skin for correct optimization and avoiding skin overdosage.

For all cT1-T2 N1 tumors, patients will be treated with concurrent radio-chemotherapy [49–51]. Selected patients with a “small” positive node (I.E., centimetric) or having an absolute contra-indication to chemotherapy may be treated with radiotherapy alone.

In the case of concomitant chemotherapy, the selection, delineation and prescription will follow the same principles as stated above, except that.

- Nodal Gross Tumor Volume (GTVn) will be delineated according to the clinical examination and available imaging. A 5 mm margin adapted to anatomical barriers will be added to generate the nodal Clinical Target Volume treated (CTVn), to be treated at therapeutic dose.
- Only normo-fractionated schedule may be used but may be accelerated at the discretion of the center by use of 6 fractions a week.
- The standard chemotherapy regimen is the Cisplatin 100 mg/m^2^ on days 1 – 22 and 43 (5 fractions a week) or on days 1 and 22 (6 fractions a week). In case of contra-indication to cisplatin, alternatively, carboplatin-5FU may be administered on weeks 1, 4 and 7 (5 fractions a week) or on weeks 1 and 4 (6 fractions a week). Weekly schedules or cetuximab or any other investigational or non-investigational radio-sensitizing drugs are not allowed.

#### Radiotherapy quality assurance

Before authorization and inclusion to participate in this study, each center must perform a Dummy Run. This consists to a delineation and planning exercise according to the protocol in a provided patient case. This will be reviewed by our radiotherapy expertise committee.

After inclusion, the three first patients must be submitted for central review of the planned treatment and delineation before radiotherapy starting. After that, randomly selected cases will be reviewed on request of the radiotherapy expertise committee.

#### Arm 2 and 3: Trans-oral Surgeries

Trans-oral surgeries can be performed by TLM or by TORS. The choice of the technique will be made during the local MTB and depending the local expertise.

Surgery must be performed within the 4 weeks after pathological confirmation of SSCC with primary tumor biopsy.

A good surgical field exposure is mandatory. The use of an appropriate mouth gag is recommended. The exposure will be evaluated before the main surgery during a rigid panendoscopy. In case of doubt, the mouth gag needs to be tested during this procedure.

- 1/ Trans-oral laser microsurgery (TLM).

A microscope with a CO_2_ laser will be used for the resection and hemostasis.

The best adapted laryngoscope to the patient anatomy should be used for an adequate exposition.

The hemostasis can be performed by a monopolar of the use of clips.

During the procedure, the superior laryngeal arteries should be ligated or clipped.

- 2/ Trans-oral robotic surgery (TORS).

TORS will be carried out using the Da Vinci Surgical robot (Intuitive Surgical, CA, USA). Surgeons on the main console and at the bedside should have adequate training and an experience of minimum 20 cases. The spatula cautery will be used to remove the tumor and perform hemostasis at the same time. The hemostasis and bleeding prevention should be made with the bipolar or using clips. During the procedure, the superior laryngeal arteries should be ligated or clipped.

- 3/ Handling of Surgical Margins.

The surgeon should try to achieve a minimum of 1 cm gross visual margins with > 3 mm microscopic margins. The resection needs to completely remove the tumor and *en-bloc* resection is preferred. The resection will be performed according the European Laryngological Society classification of endoscopic supraglottic laryngectomy [53]. In the TLM, the piecemeal resection is sometimes necessary to obtain adequate deep section margins. In this case, all the pieces of the specimen must be oriented and pinned carefully on the cork-board. The help of a schematic anatomical view is mandatory.

During the surgery, circumferential margins will be evaluated by frozen section peroperative analysis. These margins will be oriented as followed: superior, inferior, lateral, medial and deep. If needed, further resections will be performed until negative margins are obtain. The resected specimen should be oriented with the help of a schematic view on the cork-board.

Definition of the margins [54, 55]:

- Clear margins (R0): when margins are > 3 mm on the final pathology specimen
- Close margins: when they are between 1 and 3 mm.
- Positive margins (R1) when they are < 1 mm

If a positive (R1) or close margin is found on the final pathology analysis, an attempt to clear the margin may be performed within 2 weeks after the original resection in order to obtain an R0-status with the help of a new trans-oral resection in the closed/positive margins area. If clear margins cannot be obtained, postoperative radiotherapy ± chemotherapy will be administered (see below).

- 4/ Neck dissection.

In both surgical arms (Arms 2 and 3), all patients will undergo standard elective neck dissections (END) for the lymph node areas at risk on the same day of the main surgery, or differed for a maximum of 2 weeks, at the discretion of the surgeon [25]. In case of a cN0 neck, END will be limited to the levels II – III and IV. In case of a cN1 neck, and when the levels I or V are involved, the neck dissection will include these levels.

About the side:

- any lesion more than 5 mm away from the midline, a unilateral same side END is sufficient.
- for lesions closer or equal than 5 mm to the midline, a bilateral END will be done.
- The END should be oriented or separately partitioned in order to identify the level of the lymph involved on the final pathology [56–58].

- 5/ Reconstruction.

Healing of the defect is generally by secondary intention. In some rare cases, a primary closure is performed by trans-oral approach. In these T1 and T2 cases, the reconstruction with flaps can generally be avoided [25].

In case of complications likes communication between the neck dissection site and the tumor resection bed or pharyngo-cutaneous fistula, a reconstruction with flaps can be used. The choice of the subtype of flaps (pectoralis; forearm free-flap, anterolateral thigh free-flap, etc.) is left at the discretion of the surgeon or the plastic surgeon and depends on his expertise.

- 6/ Post-operative care.

Extubation: can be performed the same day or the day after the surgery, at the surgeons’ and anesthetists’ discretion [59]. A tracheostomy should typically not be required but can be performed in case of bleeding or edema risk. The need for a protective tracheostomy is judged by the surgeons and is not considered as a complication if it’s performed on the same day as the surgery. The use of a tracheostomy should also be collected.

The use of nasogastric tube or gastrostomy: is necessary at the end of the procedure. The choice of the type of interventions is left at the discretion of the surgeon and depends of the patient swallowing capabilities before the surgery. The use of nasogastric tube is preferred. The number of days and the type of support should be collected in the study [60].

In case of bleeding after the surgery: the management needs to be optimal and the safest for the patient [61]. If a surgical revision must be performed, the trans-oral approach should be preferred. The hemostasis can be done by vessels clipping, bipolar or monopolar-suction device, depending of the experience of the surgeon. If it is not sufficient, an external neck ligation of the superior laryngeal artery or the superior thyroid artery can be needed. A preventive tracheostomy can be achieved to protect the upper airways. The need of a transfusion can also be considered especially for patients with a cardiac problem history.

#### Therapy response summary (Fig. 2, Trial design)

For the Radiotherapy arm (Arm 1), the therapy response will be evaluated by the same modality as for staging (CT, MRI or PET-CT) 12–16 weeks after treatment, depending on local institutional standards. All patients must be discussed at the MTB with this imaging and clinical evaluation.

- In case of negative imaging, the patient will start the follow-up period of 2 years.
- In case of positive imaging, a panendoscopy with biopsy should be performed to confirm the residual/recurrence disease. Salvage surgery is recommended for residual or recurrent disease. For patients with residual nodes > 1 cm in size (smallest diameter), an ipsilateral neck dissection will be done. For patient with local residual or recurrent disease (at any time subsequent to radiation treatment), surgical salvage will be offered if feasible. The salvage surgery will be done by open (Total Laryngectomy) or transoral approach at the discretion of the treating surgeon and depending of the clinical situation.

For the surgical arms (Arms 2 and 3), all patients must be discussed in the MTB with the post-operative histological results. All volumes are drawn based on pre-op imaging.

Post-operative adjuvant radiotherapy alone will be performed in the presence of adverse pathological factors [32]:

- (a) close margins (1-3 mm);
- (b) peri-neural infiltration or lympho-vascular invasion.
- (c) pT3 disease.
- (d) 1 invaded lymph node > 3 cm (pN2a) or more than 2 invaded lymph nodes per side of the neck (pN2b).

The therapeutic CTV will consist of the primary tumor bed expanded in function of the quality of the surgical margins, and the eventual positive nodal levels. The elective CTV will be selected according to the international guidelines [62]. All CTVs will be expanded by an isotropic 3–5 mm margin to generate the related Planning Target Volume (PTV).

Therapeutic/elective doses will be prescribed to the respective PTV’s at 60/54 Gy in 30 fractions of 2/1,8 Gy and planned with SIB-IMRT.

Post-operative adjuvant chemo-radiation will be performed in case of [32].

- positive surgical margins in the definitive histology (< 1 mm) and no surgical revision possible;
- extracapsular extension in node(s).

Delineation of CTVs and PTVs will follow the same rules. Therapeutic/elective doses will be prescribed to the respective PTVs at 66/56,1 Gy in 33 fractions of 2/1,7 Gy and planned with SIB-IMRT. Concomitant radio-sensitizing chemotherapy with 3-weekly Cisplatin or Carboplatin-5FU will be prescribed following the same rules as for definitive-radiation.

In both cases, every reasonable effort should be made to start the post-operative (chemo)radiation within the 6 weeks after surgery.

In case of recurrence or secondary primaries, a new panendoscopy with biopsy must be performed and the results should be discussed at the MTB. In case of a very small recurrence of secondary primary, a new trans-oral procedure can be performed. In case of a bigger tumor, salvage radiotherapy should be preferred. In case of contra-indication to radiotherapy (e.g., previous radiation on the neck), a total laryngectomy or total pharyngo-laryngectomy is required.

## Statistical analysis

The primary outcome is the total MDADI scores at 12 months between the 3 arms. Assuming that the MDADI total score is normally distributed with a standard deviation of 12 points and considering a minimal clinical difference of 10 points [63], a sample size of 30 patients per arm with a confidence of 95% and a power of 90% is sufficient to detect a clinically significant difference [64–66]. Assuming a 20% of patient’s dropout, we need to recruit 36 patients per arm (total 108).

*The baseline characteristics* of the patients in the 3 arms will be described, including demographics and tumors measures at imaging, using descriptive statistics.

Differences will be assessed with a Tukey Test for K means or similar, and Chi-square tests for counts and frequencies as required.

Any difference in patient baseline heterogeneity between the arms will be assessed by the Charlson Comorbidity Index.

If a mismatch between the 3 groups in the baseline variables is discovered; a multivariate propensity scores analysis (PSA) will be undertaken to establish conditional randomness and comparability [67, 68].

*Survival rates* will be calculated from the date of MTB until the last available follow-up date or study cut-off at 2 years using the Kaplan–Meier method with differences compared using the log-rank test.

A Cox multivariate regression analysis will be used to determine baseline factors predictive of survival.

*MDADI, QLQ-C30 and H&N43 subscales* and single-item sub-scores will be summarized by means (standard deviation) and median for each trial arm and presented graphically using boxplots by trial arm and time period. The scores of the different scales will be compared between the 3 arms using mixed models for repeated measures.

### Modelling analysis

In addition to the study protocol, we will also carry out a Mathematical MultiState-transition Markov Model to perform long-term cost-effectiveness extrapolation. It will be used in order to extend the time horizon (to a life-time horizon) and to extrapolate the intermediate outcome parameters (e.g., OS and DFS) to outcome parameters (e.g., mortality). Sources will be this trial and a systemic literature review on the outcome parameters as well as the costs from the baseline and beyond the trial horizon. The model design will be a Markov Multistate Cohort Model [69, 70]. To test the robustness of uncertain parameters will be presented with one- and two-way and probabilistic sensitivity analyses (by Monte-Carlo PSA) [71]. Through worst-case scenarios and distributions of the input variables the robustness of the model will be demonstrated with regard to input.

### Why not using randomization?

First, in these small selected tumors group, patient cancer stage and tumor characteristics should be comparable (see inclusion criteria) but the influence of patient preferences is as important as the particular expertise of the individual treating medical team. Ignoring patient preferences can lead to recruitment problems, as shown in a recent study [27].

Second, the final treatment depends on the MTB decision based on the NCCN and European guidelines [31, 32] as well as the patient preference. In addition, before trial inclusion, a questionnaire will be filled in to highlight if a specific criterion has influenced the final treatment choice. We also chose to perform an observational trial to be as much possible close to real life practice (and avoiding cost linked to the randomization).

Finally, to minimize a potential center effect, patient’s inclusion is restricted to hospitals with an expertise in at least 2 different treatments modalities and treating minimum 50 cases/year. Moreover, a fixed center effect will be integrated in the statistical analysis.

## Discussion

Currently, the standard of care for small SSCC is radiotherapy, which is highly effective on locoregional tumor control but can be associated with late adverse event and toxicity such as dysphagia.

Since the development of new trans-oral technique and especially TORS, functional results are encouraging, but because of the lack of hindsight with this emerging technique, a critical analysis needs to be performed before supporting a change in the treatment strategy.

It is therefore imperative to develop treatment strategies that optimize the functional outcomes and QoL of these patients. In the current literature, no prospective head-to-head trials are available to compare objectively these two issues.

So, the main objective of this research is to assess and compare objectively and prospectively the efficacy of these therapeutic approaches in early SSCC patients according to 4 classes of outcomes; quality of life, oncological, functional and economic resources.

Regarding QoL questionnaires, the MDADI and the EORTC H1N43 will allow to specifically assess dysphagia and to compare our results with those of the current literature.

The CTC-AE toxicity questionnaire will be evaluated by the physician to objectively report on the early and late toxicities.

The impact on the global QoL is evaluated with the EORTC QLQ-C30 questionnaire.

**Fig. 4:**
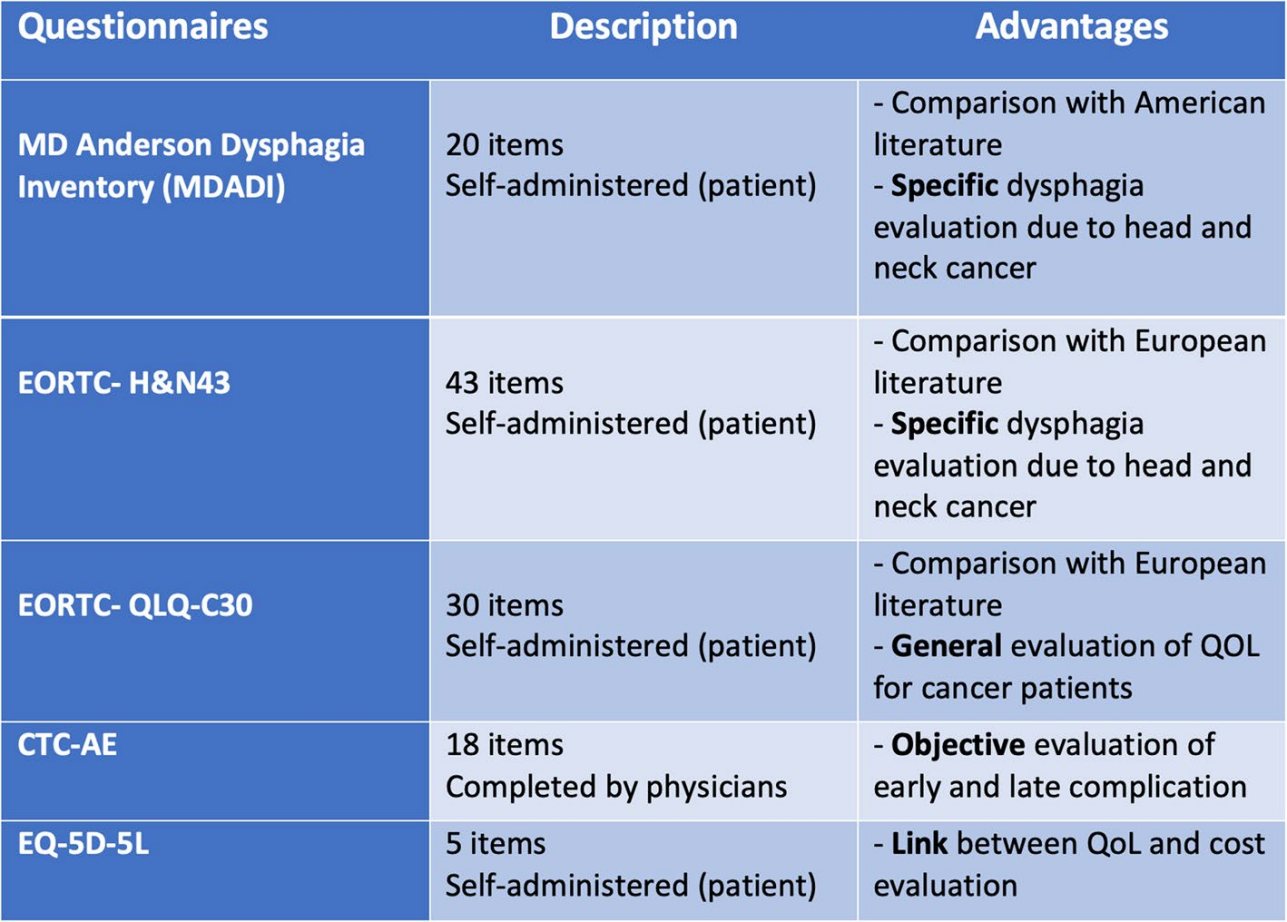
QoL questionnaires comparison

Finally, the EQ-5D-5L QoL questionnaire is used to make a link between the QoL outcomes by calculating the utilities, and also QALYs, and the associated costs (the different QoL questionnaires and their advantages are summarized in Fig. 4).

## Data Availability

The datasets used and/or analyzed during the current study are available from the corresponding author on reasonable request.

## Abbreviations

CTC-AE: Common Terminology Criteria for Adverse Events
CTV: Clinical Target Volume
DSS: Disease Specific Survival
FEES: Fiberoptic Endoscopic Evaluation of Swallowing
GTV: Gross Tumor Volume
IMRT: Intensity Modulated RadioTherapy
MDADI: MD Anderson Dysphagia Index
MTB: Multidisciplinary Tumor Board
NCCN: National Comprehensive Cancer Network
OS: Overall survival
PSA: Propensity Score Analysis
PTV: Planning Target Volume
QoL: Quality of Life
RCT: Radio Chemo-Therapy
SIB: Simultaneous Integrated Boost
SSCC: Supraglottic Squamous Cell Carcinoma
TLM: Transoral Laser Microsurgery
TORS: Trans-Oral Robotic Surgery
